# *NTHL1* biallelic mutations seldom cause colorectal cancer, serrated polyposis or a multi-tumor phenotype, in absence of colorectal adenomas

**DOI:** 10.1038/s41598-019-45281-1

**Published:** 2019-06-21

**Authors:** Sami Belhadj, Isabel Quintana, Pilar Mur, Pau M. Munoz-Torres, M. Henar Alonso, Matilde Navarro, Mariona Terradas, Virginia Piñol, Joan Brunet, Victor Moreno, Conxi Lázaro, Gabriel Capellá, Laura Valle

**Affiliations:** 1 Hereditary Cancer Program, Catalan Institute of Oncology, IDIBELL, 08908 Hospitalet de Llobregat, Barcelona, Spain; 2 Program in Molecular Mechanisms and Experimental Therapy in Oncology (Oncobell), IDIBELL, 08908 Hospitalet de Llobregat, Barcelona, Spain; 3 Centro de Investigación Biomédica en Red de Cáncer (CIBERONC), Madrid, Spain; 4 Unit of Biomarkers and Susceptibility, Cancer Prevention and Control Program, Catalan Institute of Oncology, IDIBELL, Barcelona, Spain; 5 Centro de Investigación Biomédica en Red de Epidemiologia y Salud Pública (CIBERESP), Madrid, Spain; 6 Department of Clinical Sciences, Faculty of Medicine, University of Barcelona, Barcelona, Spain; 7 Gastroenterology Unit, Hospital Universitario de Girona Dr Josep Trueta, 17007 Girona, Spain; 8 School of Medicine, University of Girona, 17071 Girona, Spain; 9 Hereditary Cancer Program, Catalan Institute of Oncology, IDIBGi, 17007 Girona, Spain

**Keywords:** Cancer genetics, Genetic counselling, Colorectal cancer

## Abstract

The cancer-predisposing syndrome caused by biallelic mutations in *NTHL1* may not be a solely colorectal cancer (CRC) and polyposis syndrome but rather a multi-tumor recessive disease. The presence of ≤10 adenomas in several mutation carriers suggests a possible causal role of *NTHL1* in hereditary or early-onset nonpolyposis CRC. The involvement of *NTHL1* in serrated/hyperplastic polyposis remains unexplored. The aim of our study is to elucidate the role of *NTHL1* in the predisposition to personal or familial history of multiple tumor types, familial/early-onset nonpolyposis CRC, and serrated polyposis. *NTHL1* mutational screening was performed in 312 cancer patients with personal or family history of multiple tumor types, 488 with hereditary nonpolyposis CRC, and 96 with serrated/hyperplastic polyposis. While no biallelic mutation carriers were identified in patients with personal and/or family history of multiple tumor types or with serrated polyposis, one was identified among the 488 nonpolyposis CRC patients. The carrier of c.268C>T (p.Q90*) and 550-1G>A was diagnosed with CRC and meningioma at ages 37 and 45 respectively, being reclassified as attenuated adenomatous polyposis after the cumulative detection of 26 adenomas. Our findings suggest that biallelic mutations in *NTHL1* rarely cause CRC, a personal/familial multi-tumor history, or serrated polyposis, in absence of adenomas.

## Introduction

In 2015 Weren *et al*. described a hereditary cancer syndrome caused by biallelic mutations in the DNA base excision repair gene *NTHL1*, characterized by attenuated adenomatous polyposis and increased colorectal cancer (CRC) risk, largely resembling the recessive syndrome caused by *MUTYH* mutations^1^. To date, 33 homozygous or compound heterozygous *NTHL1* mutation carriers have been reported (21 families)^1–8^. More than 5 colonic adenomas (range: 6 to >50) were identified in 24 of the 28 (85%) mutation carriers who underwent colonoscopy screening, and CRC was diagnosed in 19 (68%) of them. Noteworthy, 17 carriers (57%) were diagnosed with multiple primary malignant tumors in extracolonic locations, being the most recurrently found breast and endometrial tumors, head neck squamous cell carcimomas, meningiomas, and bladder and basal cell carcinomas, suggesting that the *NTHL1*-associated syndrome is a multi-tumor disease rather than a solely CRC syndrome. On the other hand, the fact that at least ¼ (7/28) of the reported biallelic mutation carriers who underwent colonoscopy screening had ≤10 adenomas, and that ≥5 hyperplastic polyps were detected in five carriers (polyp number range: 5–>30), lead us to suspect a possible association of *NTHL1* mutations with nonpolyposis CRC and serrated/hyperplastic polyposis.

Based on previous evidence and with the aim of refining the phenotypic characteristics of the *NTHL1*-associated syndrome, here we evaluated the implication of *NTHL1* biallelic mutations in the predisposition to personal or familial history of multiple tumor types, familial/early-onset nonpolyposis CRC, and serrated/hyperplastic polyposis.

## Materials and Methods

### Patients

In order to evaluate the role of *NTHL1* biallelic mutations in the context of a multi-tumor syndrome, we studied 312 unrelated cancer-affected patients who fulfilled the selection criteria indicated in Supplementary Table 1 and without mutations in the high penetrance genes associated with the corresponding phenotypes. Briefly, we included cancer-affected individuals with personal or familial history of: (i) colorectal, endometrial, small intestine or gastric cancer AND breast or ovarian cancer (n = 122); (ii) brain cancer AND any other tumor (n = 22); (iii) breast, endometrial, brain or skin cancer AND > 5 adenomatous or hyperplastic polyps (classic and attenuated familial adenomatous polyposis excluded) (n = 34); (iv) multiple primary tumors (excluding the combinations indicated in the previous categories) (n = 30); and (v) patients fulfilling the clinical criteria for germline *TP53* mutational screening but with no mutations in the gene (Li-Fraumeni) (n = 104).

A total of 488 hereditary nonpolyposis CRC cases (473 families) were also included in the study^5^. Nonpolyposis cases were mismatch repair (MMR)-proficient, i.e., their tumors showed microsatellite stability and expression of the MMR proteins MLH1, MSH2, MSH6, and PMS2. All tested individuals were affected with cancer, 96.3% with CRC. The mean age at cancer diagnosis was 49 (range: 16–82). Among the 473 studied families, 58 (12.2%) fulfilled the Amsterdam criteria, 385 (81.4%) the Bethesda guidelines, and 30 (6.3%), none the established criteria for hereditary nonpolyposis CRC. Detailed description of the hereditary nonpolyposis CRC cases is shown in Supplementary Table 2.

Also, 96 individuals diagnosed with hyperplastic/serrated polyposis^9^, were screened for germline mutations in *NTHL1*. Description of this cohort is shown in Supplementary Table 3.

All patients were assessed at the Hereditary Cancer Program of the Catalan Institute of Oncology (Spain) between 1999 and 2017. Informed consent was obtained from the participants and all methods were performed in accordance with relevant guidelines and regulations. The study received the approval of the Ethics Committee of the Institut d’Investigació Biomèdica de Bellvitge (IDIBELL).

### Mutational screening of *NTHL1*

*NTHL1* c.268C > T (p.Q90*) was genotyped in all patients by using a KASP genotyping assay (LGC Genomics, Hoddesdon, UK) in a LightCycler® 480 system (Roche, Basel, Switzerland).

Mutational screening of protein-coding exons and flanking sequences (+/−20 base pairs) was performed by direct automated (Sanger) sequencing in the 312 cancer patients with familial/personal history of multiple tumor types and in the 96 individuals with hyperplastic/serrated polyposis. Sequencing was performed at STAB VIDA (Caparica, Portugal) and data was analyzed with SeqMan Pro (Lasergene 13, DNASTAR, Madison, WI) and/or Mutation Surveyor v.3.10 (SoftGenetics, State College, PA). The primers used for amplification and sequencing are shown in Supplementary Table 4.

Hereditary nonpolyposis CRC patients were screened for *NTHL1* mutations using a combination of PCR amplification in pooled DNAs and targeted massively parallel sequencing, as previously described^10^, using the amplification primers listed in Supplementary Table 4. Sanger sequencing of the affected exon was performed to identify the mutated individuals among the samples included in the corresponding DNA pool.

### *In silico* predictions

The impact of missense variants at the protein level was analyzed using the *in silico* algorithms PolyPhen-2, SIFT, CONDEL, Mutation Taster and Align GVGD^11–15^. The potential effects on splicing were evaluated by using Human Splice Finder v.3.0^16^. Except for CONDEL, prediction data were provided by Alamut Visual v2.7.1 (Interactive Biosoftware, Rouen, France).

### PCR amplicon cloning

Exons 2–4 (2,985 bp) were PCR amplified in the gDNA extracted from the carrier of *NTHL1* c.268C>T and c.550-1G>A, cloned into the pGEM-T Easy Vector System (Promega, Madison, Wisconsin, USA) and transformed into JM109 competent cells (Promega). Individual colonies were isolated, PCR amplified and sequenced.

### *NTHL1* constitutional methylation

Methylation of the promoter of *NTHL1* was analyzed by means of methylation-specific melting curve analysis (MS-MCA) on bisulfite-modified DNA (EZ DNA Methylation-Gold Kit, Zymo Research, Orange, CA, USA). Thirty-eight and 17 CpG sites located in the *NTHL1* promoter region were evaluated in two independent assays. Methylation status was determined comparing melting curves obtained from samples versus controls, which included the CpG methylated Jurkat genomic DNA as methylated control, and DNA amplified by whole genome amplification as non-methylated control. Primer sequences are shown in Supplementary Table 4.

### *NTHL1* copy number alterations

Assessment of copy number variation in the *NTHL1* region was carried out using the Illumina Infinium Global Screening Array v2.0. Data on hybridization intensity for each probe were used to calculate the Log R ratio (LRR) and B allele frequency (BAF) values. These values were used to identify copy number regions and to segment the genome. Segmentation calculations were performed with genoCN software^17^ using the R package.

## Results and Discussion

No biallelic *NTHL1* mutation carriers were identified among the 312 patients with familial/personal history of multiple tumor types (polyposis and patients fulfilling the clinical criteria for hereditary nonpolyposis CRC cancer, were excluded). The c.268C>T (p.Q90*) recurrent pathogenic mutation, c.444G>A (p.A148=) and c.527 T>C (p.I176T), were identified in heterozygosis in 3 unrelated patients (Supplementary Table 5).

*NTHL1* mutational screening in the 488 MMR-proficient hereditary colorectal cancer patients (473 families), which included 58 families fulfilling the Amsterdam criteria, identified one compound heterozygous of c.268C>T (p.Q90*) and c.550-1G>A (Table 1). No additional relatives were available for segregation analysis. Cloning of the PCR product that included the two variants confirmed the biallelic nature of the mutations; i.e., they were found to be in different alleles, in *trans* (Supplementary Fig. 1). In addition to the biallelic mutation carrier, one heterozygous carrier of c.268C>T (p.Q90*), one of c.550-1G>A, one of c.793G>A (p.A265T) and two of c.527T>C (p.I176T), were identified (Supplementary Table 6). *NTHL1* was re-sequenced in the five heterozygotes in order to assess the presence of a second mutation, but none was detected. The two carriers of c.268C>T (p.Q90*) in the MMR-proficient nonpolyposis CRC series had been previously identified by our group by mutation-specific genotyping^5^. However, in that study, sequencing of the complete coding region of *NTHL1* in the two carriers failed to identify in one of them the herein reported second *NTHL1* mutation, c.550-1G>A, possibly due to allele dropout.

**Table 1:** *NTHL1* variants identified in the studied cohorts in biallelic state.

*NTHL1* variant (NM_002528.5)	Nature	dbSNP	HSF 3.0	Population MAF (GnomAD)	Classification
c.268C>T; p.G90*	Stop gain	rs150766139	n.a.	0.14% (0.19% Eur)	Pathogenic
c.550-1G>A	Splice site	rs779757251	Altered WT acceptor site	0.0086% (0.009% Eur)	Pathogenic

Abbreviations: Eur, European non-Finnish population; GnomAD, Genome Aggregation Database; HSF 3.0, Human Splicing Finder v.3.0; MAF, minor allele frequency; n.a., not available information; WT, wildtype.

**Figure 1:**
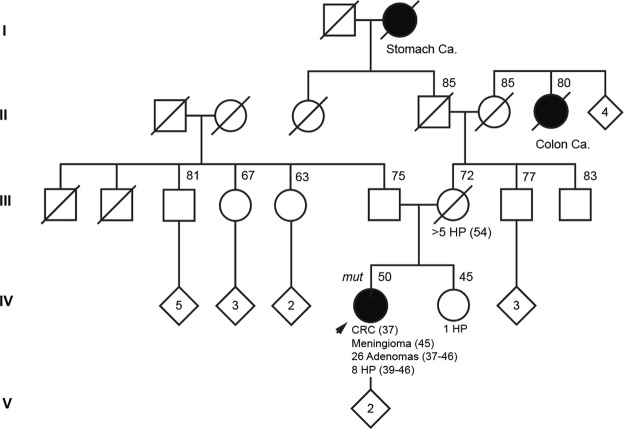
Pedigree of the carrier of *NTHL1* c.[268C>T];[550-1G>A]. Filled black symbol, cancer affected. The black arrow is pointing out the index case. Ages at information gathering or at death, when available, are indicated on the top-right corner, and ages at cancer diagnosis, after tumour type. Abbreviations: ca, cancer; CRC, colorectal cancer; HP, hyperplastic polyps; mut, *NTHL1* biallelic carrier.

The biallelic carrier of the *NTHL1* pathogenic mutations c.[268C>T]; [550-1G>A] was diagnosed with CRC and 3 adenomas at age 37, thus fulfilling the Bethesda criteria for hereditary CRC. Review of her updated clinical history revealed that annual/biennial colonoscopy screenings between ages 37–46 (8 colonoscopies) detected a total of 26 adenomas and 8 hyperplastic polyps, which now leads to a reclassification as attenuated polyposis. Moreover, she was diagnosed with a meningioma at 45 years of age (Family pedigree in Fig. 1).

Broderick *et al*. analyzed *NTHL1* from whole-exome sequencing data performed in 863 unexplained familial/early-onset CRC cases, finding no biallelic mutations in nonpolyposis CRC patients (biallelic *NTHL1* mutations were identified in a 41 year-old patient diagnosed with CRC and polyposis)^3^. Likewise, Zhang *et al*. sequenced the *NTHL1* gene in 140 patients diagnosed with CRC before 35 years of age (from an unselected series of >2,300 Chinese CRC patients), finding no pathogenic mutations in the gene^18^. The additive evidence gathered in these two recent studies and ours (total number of patients studied: 1,491; number of *NTHL1* biallelic carriers: 1, who has been reclassified as having attenuated adenomatous polyposis) suggests that the contribution of biallelic *NTHL1* mutations to nonpolyposis CRC is null or, at most, negligible.

On the other hand, no *NTHL1* mutations were identified among the 96 serrated polyposis patients, either in homozygosis or heterozygosity. Although our findings need to be validated in larger cohorts, they suggest that biallelic *NTHL1* mutations are not a major cause of serrated polyposis, in spite of the detection of several hyperplastic polyps (range: 5->30) in 6 out of 34 *NTHL1* biallelic mutation carriers reported to date, including the c.[268C>T]; [c.550-1G>A] carrier identified in the current study, with 26 adenomas and 8 hyperplastic polyps detected before age 50.

In the herein studied cohorts, the frequency of heterozygous missense, splice-site and loss-of-function variants (population minor allele frequency (MAF) < 1%) in *NTHL1* (NM_002528.5): 3/312 (0.96%) in patients with personal and/or familial history of multiple tumor types, 5/487 (1.03%) (5/472 (1.06%) if only unrelated patients are considered) in hereditary nonpolyposis CRC - the *NTHL1*-mutated attenuated adenomatous polyposis patient excluded-, and 0/96 (0%) in hyperplastic/serrated polyposis, is not higher than the frequency identified in European cancer-free population, i.e. 1.74% (75/4299 individuals; source: NHLBI GO Exome Sequencing Project http://evs.gs.washington.edu/EVS/), *a priori* supporting the lack of association of monoallelic *NTHL1* mutations with cancer predisposition.

Although never reported before, we assessed the presence of *NTHL1* promoter hypermethylation in the identified monoallelic mutation carriers in order to discard the presence of other types of alterations affecting the -*a priori*- wildtype allele. None of the eight heterozygotes showed constitutional *NTHL1* CpG island methylation (Supplementary Fig. 2). Copy number alteration analysis could be performed in six of the eight monoallelic mutation carriers identified, finding no large deletions or rearrangements affecting the gene. Of note, neither our study nor previous analyses reported so far have included the study of large deletions or constitutional epimutations in *NTHL1* in the complete series, which might have caused the under-detection of mutations in the studied cohorts.

In conclusion, our findings suggest that: (i) the *NTHL1*-associated syndrome is not a multi-tumor syndrome in absence of adenomatous polyposis, (ii) the serrated polyposis syndrome is not caused by *NTHL1* mutations, and (iii) the presence of biallelic *NTHL1* mutation carriers in hereditary CRC patients without polyposis is, if any, extremely rare.

## Supplementary information

Supplementary Information
