# Supplementary material for: NTHL1 biallelic mutations seldom cause colorectal cancer, serrated polyposis or a multi-tumor phenotype, in absence of colorectal adenomas
Source: Sci Rep. 2019 Jun 21;9:9020. doi: 10.1038/s41598-019-45281-1 (PMC6588610; doi:10.1038/s41598-019-45281-1)
Supplement: Supplementary file 1 — Supplementary Information [file 41598_2019_45281_MOESM1_ESM.pdf]

NTHL1 biallelic mutations seldom cause colorectal cancer, serrated polyposis or a multi-tumor phenotype, in absence of colorectal adenomas

Sami Belhadj, Isabel Quintana, Pilar Mur, Pau M. Munoz-Torres, M. Henar Alonso, Matilde Navarro, Mariona Terradas, Virginia Piñol, Joan Brunet, Victor Moreno, Conxi Lázaro, Gabriel Capellá, Laura Valle*.

The first two authors contributed equally to this work

**Corresponding author.*

SUPPLEMENTARY INFORMATION

Supplementary Table 1. Description of the groups of cancer-affected unrelated patients with personal or familial history of multiple tumor types included in the study.

Selection criteria	No. patients (total: 312)
[a]**Personal and/or family history of:** CRC, endometrial, small intestine or gastric cancer & breast or ovarian cancer	122
**Personal and/or family history of:** Brain cancer & any other tumour	22
[b]**Personal and/or family history of:** Breast, endometrial, brain or skin cancer & >5 polyps	34
**Personal and/or family history of:** Multiple primary tumours (excluding the tumour combinations included in the other categories)	30
**Patients/families with no germline *TP53* mutation fulfilling the following criteria**[1]:	104
Classic Li-Fraumeni (n=3)	
Li-Faumeni-like (n=15)	
Chompret / Revised Chompret (n=61)	
Eeles[2] (n=25)	

a. Families/patients fulfilling the classical criteria for HNPCC (Amsterdam or Bethesda) were not included in this study.
b. The polyps were either adenomas or hyperplastic polyps. Classic/attenuated colonic adenomatous polyposes had been previously studied,[3] and were not included in this study.

Supplementary Table 2. Description of the non-polyposis CRC cases (n=488 patients, 473 families) included in the study.

	No. families	No. patients	Mean age at cancer diagnosis	Median age at cancer diagnosis	Age range
**TOTAL**	**473**	**488**	**49.0**	**47**	**16-82**
**CRC-affected proband**					
Amsterdam [I / II]	55 [39 / 16]	57 [41 / 16]	47.8	46	19-78
Bethesda	384	397	49.1	47	16-82
No HNPCC criteria	16	16	57.7	58	50-69
Subtotal	455	470	49.0	47	16-82
**Ovarian cancer-affected proband**					
Amsterdam [I / II]	1 [0 / 1]	1 [0 / 1]	58	58	-
No HNPCC criteria	8	8	40.5	40	24-63
Subtotal	9	9	41	41	24-63
**Gastric cancer-affected proband**					
Amsterdam [I / II]	2 [0 / 2]	2 [0 / 2]	45	45	39-51
No HNPCC criteria	4	4	41.25	41	34-49
Subtotal	6	6	42.5	41	34-51
**Other tumors**					
Bethesda	1	[a]1			
No HNPCC criteria	2	[b]2			
Subtotal	3	3	49.1		30-59

a. Patient: Renal pelvis tumor and pancreatic cancer at age 59.
b. Patient 1: Endometrial cancer at age 30; Patient 2: Melanoma at age 35 and pancreatic cancer at age 62.

Supplementary Table 3. Description of the serrated/hyperplastic polyposis cohort (n=96) included in the study.

Characteristics	Mean (range)
**Age at polyposis diagnosis**	51.8 years (8-73)
**Total number of polyps**	39 (3-195)
**No. hyperplastic and serrated polyps**	28.6 (3-163)
**No. adenomatous polyps**	3.9 (0-65)
**No. patients with histologic report of serrated polyps (n; %)**	45; 46.9%
	**# cases (%)**
**Gender**	Male: 62 (64.6%)<br>Female: 34 (35.4%)
**CRC diagnosis**	36 (37.5%)<br>Mean age at diagnosis: 51.9; range: 24-75
**Family history of polyposis**	15 (15.6%)
**Cases with adenomas in addition to hyperplastic/serrated polyps**	67 (69.8%)<br>Mean number of adenomas: 6; range:1-65
***APC* gene tested**	[a]37/96 (38.5%)
***MUTYH* gene tested**	[b]45/96 (46.9%)
***RNF43* gene tested**	[c]96/96 (100%)

a. Variants of unknown significance in APC were detected in 2 individuals.
b. One *MUTYH* biallelic carrier, c.494A>G; p.(Tyr165Cys) and c.1145G>A; p.(Gly382Asp), was identified in an individual with CRC, 6 adenomas and 21 serrated polyps diagnosed at age 48.
c. *RNF43* c.394C>T (p.R132*) was detected in a woman diagnosed with CRC and >50 polyps (including serrated lesions) at age 55[4].

Supplementary Table 4. Primers used for PCR amplification and direct automated sequencing, and for promoter methylation analysis.

NTHL1 **(NM_002528; GRCh37)**	**Forward primer (5'-3')**	**Reverse primer (5'-3')**	**Amplicon size (bp)**
Exon 1	ggggtccgcctctctagg	gacagagtggtgggaaagga	690
Exon 2	atggacagttgtggcatgaa	aaaccactggtgtcctgacc	536
Exon 3	gaggctgatgcctcaagtgt	agaggaaggaagggaggatg	513
Exon 4	agcctacacgtgcatcattg	cacaggtcacaaggatgtgg	466
Exon 5-6	gctcaggtgatgggagagag	tcctgaagcgtaaagccact	611
NTHL1meth_38 CpG	ttatgatttaaaattaagggatgtt	acacaaaactacaactcccaacaaactc	306
NTHL1meth_17 CpG	atattaggtttggtttagagagttttttat	acatcccttaattttaaatcataac	207

Supplementary Table 5. Heterozygous *NTHL1* variants identified in patients with personal and/or family history of multiple tumor types. *In silico* prediction of the variants' effect, and phenotypic characteristics of the carriers.

***NTHL1* variant**	**dbSNP**	**Population MAF (GnomAD)**	***in-silico* Predictions**	**Splicing prediction (HSF 3.0)**	**Carrier phenotype (age at diagnosis)**	**Familial history of cancer/polyps**
**c.268C>T; p.Q90***	rs150766139	0.14<br>(0.19% Eur)	n.a.	n.a.	Thyroid ca. (23)	Maternal branch: Breast, renal and bone tumors<br><br>Father: Cholangiocarcinoma
[b]**c.444G>A; p.A148=**	rs768014084	0.004%<br>(0.002% Eur)	n.a.	New Acceptor Site	CRC	Paternal branch: Colon, breast, stomach and prostate tumors
**c.527T>C; p.I176T**	rs1805378	0.18%<br>(0.22% Eur)	SIFT: D (0.01)<br>PPH2: Prob. D (1.000/0.99)<br>Mutation Taster: D<br>CONDEL: D<br>[a]Align GVGD: Class C65<br>(GV: 4.86 - GD: 89.28)	No changes	Signet ring cell ovarian ca. (35)<br><br>Stomach cancer (56)	No

a. AlignGVGD: C0: least likely to interfere with function, C65: most likely to interfere with function.
b. Large genomic rearrangements in *NTHL1* were not identified.

Abbreviations: ca, cancer; CRC, colorectal cancer; D, damaging; Eur: European (non-Finnish) population; GD, Grantham deviation; GnomAD, Genome Aggregation Database; GV, Grantham variation; HSF, Human Splicing Finder v.3.0; MAF, minor allele frequency; n.a., not available information; Prob. D, probably damaging; PPH2, PolyPhen-2 score (HumDiv/HumVar).

Supplementary Table 6. Heterozygous *NTHL1* variants identified in hereditary non-polyposis CRC families. *In silico* predictions and phenotypic characteristics of the carriers.

***NTHL1* variant**	**dbSNP**	**Population MAF (GnomAD)**	***in-silico* Predictions**	**Splicing prediction (HSF 3.0)**	**HNPCC criteria**	**Carrier phenotype (age at diagnosis)**	**Familial history of cancer/polyps (age at diagnosis)**
[a,b]**c.268C>T; p.Q90***	rs150766139	0.14%<br>(0.19% Eur)	n.a.	-	Bethesda	CRC (40)	Father: CRC (66)<br>Sister: Cervix ca. (37)
[b]**c.550-1G>A**	rs779757251	0.0086%<br>(0.009% Eur)	n.a.	Altered WT acceptor site		CRC x2 (70)<br>Carcinoid tumor of cecal appendix (70)<br>[d]<20 polyps	Brother: CRC (69) & melanoma (73)
[b]**c.793G>A; p.A265T**	rs148474733	0.017%<br>(0.00079% Eur)	SIFT: N (0.3)<br>PPH2: Poss.D/ N (0.766/0.418)<br>Mutation Taster: D<br>CONDEL: D<br>[c]Align GVGD: Class C0<br>(GV: 232.67 - GD: 0.00)	Altered ESE	Bethesda	Rectum ca (20)	No
[b]**c.527T>C; p.I176T**	rs1805378	0.18%<br>(0.22% Eur)	SIFT: D (0.01)<br>PPH2: Prob. D (1.000/0.99)<br>Mutation Taster: D<br>CONDEL: D<br>[c]Align GVGD: Class C65<br>(GV: 4.86 - GD: 89.28)	No changes	Bethesda	CRC (41)	Father: Prostate ca. (82)<br>Brother: CRC (49) & 2 polyps
[b]**c.527T>C; p.I176T**	rs1805378	0.18%<br>(0.22% Eur)	SIFT: D (0.01)<br>PPH2: Prob. D (1.000/0.99)<br>Mutation Taster: D<br>CONDEL: D<br>[c]Align GVGD: Class C65<br>(GV: 4.86 - GD: 89.28)	No changes	Bethesda	CRC (31)<br>1 adenoma (32)	Father: CRC (70)<br>Mother: CRC (82)<br>Maternal aunt: Breast ca. (70)

a. Reported by Belhadj *et al*, 2017[3].
b. Large genomic rearrangements in *NTHL1* were not identified.
c. AlignGVGD: C0: least likely to interfere with function, C65: most likely to interfere with function.
d. Review of the updated clinical history revealed a cumulative number of polyps >70 in periodic colonoscopies.

Abbreviations: ca., cancer; CRC, colorectal cancer; Eur, European (non-Finnish) population; GD, Grantham deviation; GnomAD, Genome Aggregation Database; GV, Grantham variation; HNPCC, hereditary nonpolyposis colorectal cancer; D, damaging; N, neutral/tolerated; Prob. D, probably damaging; Poss. D, possibly damaging; PPH2, PolyPhen-2 score (HumDiv/HumVar); HSF, Human Splicing Finder v.3.0; ESE, Exonic splicing enhancer; MAF, minor allele frequency; WT, wildtype.

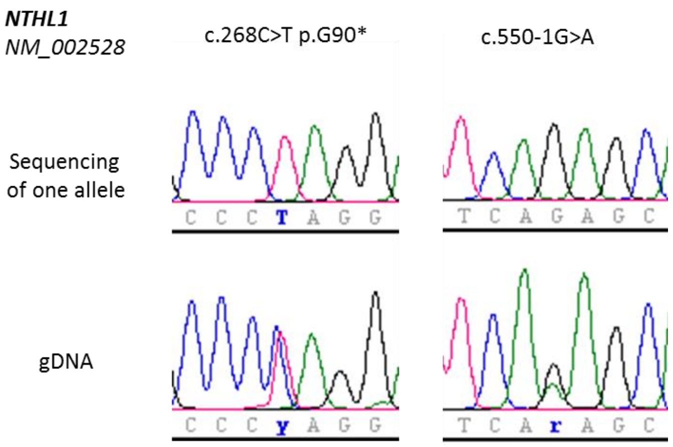

Supplementary Figure 1. Evidence indicating that *NTHL1* mutations in the compound heterozygote are in *trans* (c.[268C>T];[550-1G>A]). Sequencing of one colony containing one allele of the region comprised between exons 2 and 4 and therefore including the positions c.268 and c.550-1. The represented allele carries the c.268C>T mutation but not c.550-1G>A, which was detected in the other allele (not shown).

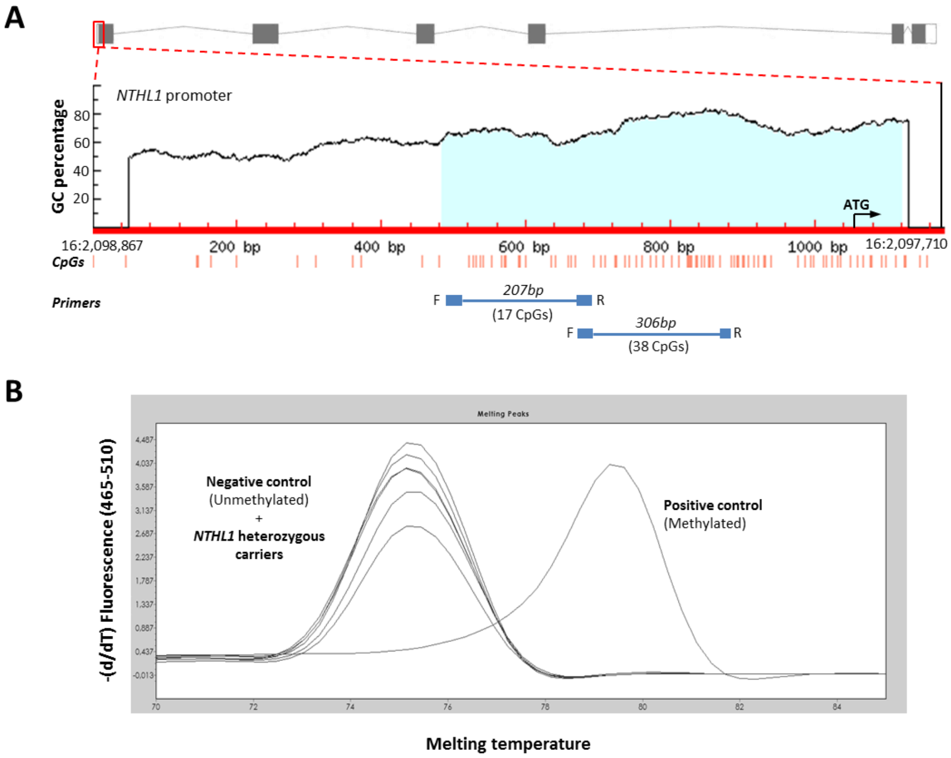

Supplementary Figure 2. Constitutional *NTHL1* promoter methylation status. A. Prediction of *NTHL1* CpG island (in blue), and location of CpG sites (red bars), and primers (blue rectangles) used for MS-MCA assays (adapted from Methprimer program[5]). The transcription start site of *NTHL1* is indicated by ATG. **B.** Melting curves results of the *NTHL1* promoter methylation analysis (17 CpG sites) by MS-MCA. Methylation was neither observed when analyzing the 38 CpG region (data not shown).

REFERENCES

1. Guha, T. & Malkin, D. Inherited TP53 Mutations and the Li-Fraumeni Syndrome. *Cold Spring Harb Perspect Med* **7** (2017).
2. Eeles, R.A. Germline mutations in the TP53 gene. *Cancer Surv* **25**, 101-24 (1995).
3. Belhadj, S. et al. Delineating the Phenotypic Spectrum of the NTHL1-Associated Polyposis. *Clin Gastroenterol Hepatol* **15**, 461-462 (2017).
4. Quintana, I. et al. Evidence suggests that germline RNF43 mutations are a rare cause of serrated polyposis. *Gut* (2018).
